# Intravaginal probiotics before embryo transfer do not improve pregnancy rates in recurrent implantation failure cases: An RCT

**DOI:** 10.18502/ijrm.v22i5.16435

**Published:** 2024-07-08

**Authors:** Mina Naghi Jafarabadi, Farnaz Hadavi, Maedeh Ahmadi, Masoumeh Masoumi, Sara Zabihzadeh

**Affiliations:** Vali-E-Asr Reproductive Health Research Center, Family Health Research Institute, Imam Khomeini Hospital Complex, Tehran University of Medical Sciences, Tehran, Iran.

**Keywords:** Assisted reproductive technique, Probiotics, Microbiota, Pregnancy rate.

## Abstract

**Background:**

Considering the considerable influence of the vaginal microbiome on endometrial receptivity and embryo implantation, we hypothesized that cases of recurrent implantation failure (RIF) might benefit from the intravaginal probiotic administration.

**Objective:**

Evaluation of the effects of intravaginal probiotic administration before frozen embryo transfer (FET) on the rates of pregnancy and the status of vaginal lactobacillary flora in cases of RIF.

**Materials and Methods:**

This was a randomized, parallel-group, clinical trial conducted at an infertility clinic in Tehran, Iran between January 2021 and September 2022. A total of 166 reproductive-aged women with a history of unexplained RIF were randomly assigned to either the probiotic group or the control group (n = 83/each group). The probiotic group received intravaginal probiotics (LactoVagⓇ) daily for 2 wk from the second day of the menstrual cycle along with the routine treatment of FET. The control group received only the routine treatment of FET. The primary outcome was the chemical pregnancy rate, and the secondary outcomes were the clinical pregnancy rate and the status of vaginal lactobacillary flora.

**Results:**

A total of 163 participants were included in the final analysis. The probiotic group had a slightly higher chemical pregnancy rate than the control group (39.02% vs. 33.33%), but the difference was not statistically significant (risk ratio: 1.71, 95% CI: 0.77–1.76; p = 0.449). The clinical pregnancy rate was also non-significantly higher in the probiotic group than the control group (37.80% vs. 33.33%; RR: 1.14, 95% CI: 0.76–1.74; p = 0.623).

**Conclusion:**

Intravaginal probiotic administration did not significantly improve the pregnancy rates in RIF cases undergoing FET. Further studies are needed to explore the optimal dose, duration, and timing of probiotic administration, as well as the mechanisms of action and the potential adverse effects of probiotics on the vaginal microbiome and the implantation process.

## 1. Introduction

Infertility, which is defined as the failure to achieve pregnancy despite having 12 months or more of regular unprotected sexual intercourse affects millions of couples worldwide (1). Assisted reproductive technologies (ART) offered a promising solution to overcome infertility and achieve pregnancy in many couples. However, not all ART cycles result in the successful implantation of the transferred embryos. Recurrent implantation failure (RIF) is one of the most challenging issues in reproductive biomedicine, as it has no clear definition, diagnosis, or treatment. Meanwhile, RIF is generally defined as the failure to achieve pregnancy after 3 or more in vitro fertilization (IVF)- embryo transfer (ET) cycles with good quality embryos and normal endometrial proliferation thickness and pattern (2).

RIF has multifactorial and poorly understood etiology, but it can involve endometrial and embryonic factors, or the interaction between them (3). Recently, the role of the microbiome of the female reproductive tract in embryo implantation has gained attention. The microbiome is the collection of microorganisms that live in and on the human body. The microbiome of the reproductive tract extends from the vagina to the fallopian tubes and influences the immune system and the microenvironment of the uterus (4). *Lactobacillus* species are the dominant bacteria in the healthy reproductive tract, and they produce various substances, such as lactic acid, bacteriocins, hydrogen peroxide, and probiotics that create a favorable microenvironment for embryo implantation (5, 6). On the other hand, the imbalance of the reproductive tract microbiome, known as dysbiosis, can lead to the overgrowth of pathogenic bacteria, such as *Gardnerella vaginalis* and *Streptococci*, and cause chronic inflammation and impairs the endometrial receptivity and the survival of the embryo, and consequently prevent successful implantation (7, 8). A comprehensive multicenter study was conducted to examine the endometrial microbiota (EM) in infertile patients undergoing IVF (9). The research focused on the correlation between endometrial receptivity and the EM in women having frozen embryo transfer (FET). It was found that an imbalanced EM, characterized by the presence of *Atopobium, Bifidobacterium, Chryseobacterium, Gardnerella, Haemophilus, Klebsiella, Neisseria, Staphylococcus, *and* Streptococcus*, correlated with negative IVF outcomes and reduced endometrial receptivity. Conversely, a high presence of *Lactobacillus* was linked to successful live births. These insights underscore the potential benefits of probiotic treatments in enhancing the endometrial receptivity and embryo implantation. Based on the existing evidence mentioned above in supporting the role of a *Lactobacillus*-dominated microbiome in reduction of proinflammatory cytokines that resulted from vaginal dysbiosis, administering probiotic bacteria before FET might be a beneficial adjunctive therapy for RIF cases (9–12). The World Health Organization defines probiotics as “live microorganisms which when administered in adequate amounts confer a health benefit on the host” (13). Various clinical studies investigating probiotic use prior to FET yielded positive results. However, based on their heterogeneous findings, there is still not enough scientific data to support the systematic use of probiotics for treating asymptomatic dysbiosis before IVF (14–16). Also, none of the previous studies have focused on RIF cases. To the best of our knowledge, this is the first randomized clinical trial to evaluate the effects of intravaginal probiotic administration on pregnancy rate and the status of vaginal lactobacillary flora, in RIF cases undergoing FET.

## 2. Materials and Methods

### Study settings

The study randomized, parallel-group, clinical trial study was conducted at Imam Khomeini hospital, affiliated to Tehran University of Medical Sciences, Tehran, Iran, in accordance with the institutional review board regulations and all applicable local regulations. After obtaining the required permissions, the requirement of the first participant was started in January 2021, and the follow-up of the last participant was done in September, 2022.

### Sample size

The sample size was calculated based on the expected difference in pregnancy rate between the probiotic and control groups of 0.15, with a power of 80% and a significance level of 0.05. The participants were randomly assigned to either the probiotic or the control group, with a 1:1 allocation ratio. The expected pregnancy rate was based on both previous studies and clinical experience. A dropout rate of 10% was assumed and the sample size was inflated accordingly. No interim analysis or stopping guidelines were planned for this trial. The following sample size formula for a 2-sided test of proportions was used: 


$$n={\left(Z_{\alpha /2}+Z_{\beta }\right)}^{2}*\left(p_{1}\left(1-p_{1}\right)+p_{2}\left(1-p_{2}\right)\right)/{\left(p_{1}-p_{2}\right)}^{2}$$

### Randomization, blinding, and concealment

After recruitment, participants were randomly allocated to intervention (probiotic) and control groups by an independent investigator using a randomly generated number sequence. The study methodologist designed the randomization list according to the block randomization method by using an online platform (https://www.sealedenvelope.com), which is widely recognized for its robust random number generation algorithms. A block size of 4 was chosen to ensure a balance in the allocation of participants to each group while maintaining the unpredictability of the assignment sequence. An example sequence of allocation within blocks is AABB-ABAB-ABBA-BBAA-BABA-BAAB. For concealment of the randomization list from all research teams involved in enrollment and assessment sealed envelopes method was used. The sealed envelopes were prepared by an independent administrator who was not involved in the recruitment or assessment of participants. Each envelope was opaque, securely sealed, and sequentially numbered to ensure the concealment of the allocation sequence. They were stored in a locked cabinet, accessible only to the study methodologist responsible for participant assignment. In this study, as control group did not receive a placebo, blinding was not possible for participants and all research teams including gynecologists; however, the embryologist, laboratory staff, and the statistician were blinded about the type of intervention in each group until the end of the study. To maintain the blinding of the embryologist, laboratory staff, and statistician, each participant's group assignment was coded with a unique identification number. The code key was safeguarded by the study methodologist and was not disclosed until the completion of the statistical analysis.

### Participants

This randomized clinical trial included 166 non-menopausal married women aged ≤ 40 yr old who met the following criteria: a) history of unexplained RIF (3 or more consecutive failed ETs with good quality embryos); b) having a normal uterine cavity, both ovaries, and adequate endometrial thickness and pattern.

The exclusion criteria were: a) vaginal bleeding; b) vaginitis; c) history of utero-cervical anomalies, endometriosis, chronic diseases, or endocrinopathies (like adrenal insufficiency or uncontrolled thyroid dysfunction); d) chromosomal abnormalities (either parents or previously transferred embryos); e) smoking; f) and taking antibiotics, vaginal medications (during the past 2 wk), or vaginal douche (during the past week). Investigation of eligibility criteria was performed by an infertility fellowship who also supervised the research process.

### Interventions

#### Interventions in each study group

In this study, participants in the intervention group received probiotics (10^9^ CFU) daily (LactoVagⓇ, Zist Takhmir Co. Tehran, Iran) (1 tablet intravaginally per day) for 2 wk along with the routine treatment of ET. Rout and duration of probiotic administration have been claimed by the manufacturer's instruction and also according to previous similar studies (9, 15–17). LactoVagⓇ is a synbiotic (probiotic + prebiotic) formulation, with different strains of Lactobacillus including Lactobacillus rhamnosus, Lactobacillus plantarum, Lactobacillus acidophilus, and Lactobacillus gasseri plus maltodextrin as a prebiotic.

The control group received only the routine ET treatment. We did not use a placebo in the control group, because we could not find a suitable intravaginal placebo that would not interfere with the endometrial preparation cycle, the vaginal flora, and normal vaginal discharge PH and color. Moreover, we wanted to assess the effect of probiotics on the pregnancy rate in asymptomatic cases, and we did not want to introduce any potential confounding factors, such as antibiotics or antifungals that could affect the vaginal microbiome or the detection of *Lactobacillus*. Therefore, we decided to compare the probiotic group with the standard care group, without any additional intervention.

#### Endometrial preparation protocol

In the present study, all participants underwent a hormone replacement therapy cycle before FET. Accordingly, exogenous estrogen supplements were used to stimulate endometrial growth and suppress folliculogenesis followed by exogenous progesterone therapy for luteal phase support. In that respect, oral Estradiol Valerate 2 mg film-coated tablets (Aburaihan Pharmaceutical Co., Tehran, Iran) were administered in a fixed dose of 6 mg per day from the second day of the menstrual cycle. During the estrogen therapy, the thickness and pattern of endometrium were checked through vaginal ultrasound. Adjustment of estradiol dosage during the hormone replacement therapy cycle was based on endometrial thickness. When endometrial thickness reached at least 6–8 mm, the luteal phase support was started with progesterone therapy. In this regard, a daily intramuscular (IM) injection of 100 mg progesterone (two 50 mg Femogex-IH IM injections, Iran Hormone Pharmaceutical Co., Tehran, Iran) was prescribed. The IM injection was prioritized over vaginal progesterone to prevent any interference with normal vaginal flora and PH. After ET, the luteal phase support was continued with micronized vaginal progesterone until confirmation of pregnancy.

#### Embryological considerations

#### 2.5.3.1. Embryo grading

Embryos were graded twice: once on the third day (68 ± 1 hr post-insemination) of development at cleavage (8-cell) stage, just before cryopreservation, and once after thawing, to assess probable cryodamage-induced changes. The grading was based on the latest guidelines for embryo grading. According to these criteria, embryos with proper age-specific number, and size of blastomeres (8 equally sized blastomeres), with < 10% fragmentation would be graded as A (18). In the present study, only cases that had grade A embryos, indicating optimal quality and viability, were included.

#### 2.5.3.2. Embryo cryopreservation protocol

Embryos were cryopreserved at the cleavage stage, aging for 3 days, using the vitrification method. For vitrification, Ravan Saze medical (Ravan Saze Co., Tehran, Iran) vitrification solutions kits, which consist of 3 solutions: equilibration, vitrification 1, and vitrification 2 solutions, were used. After dispensing embryos in each solution according to the manufacturer's instruction, embryos were loaded into the vitrification device and plunged into liquid nitrogen and stored in the cryotank.

#### 2.5.3.3. Embryo thawing protocol 

Cryopreserved embryos underwent the thawing procedure on the third day of progesterone therapy. The thawing protocol was performed using the Ravan Saze medical thawing solutions kit, which consists of 3 solutions: thawing, dilution, and washing solutions. The protocol was based on the manufacturer's instructions. After washing embryos in the last solution, they were transferred to a culture medium and incubated at 37 C until the ET procedure.

#### 2.5.3.4. ET Procedure

The ET procedure was performed with ultrasound guidance by a single infertility specialist. 2 or 3 embryos were transferred for each participant. Embryos were loaded into the soft catheter and was gently inserted through the cervix and advanced into the uterus under ultrasound guidance. The embryos were deposited in the upper third of the uterine cavity, about 1–2 cm from the fundus. The catheter was slowly withdrawn and checked for any retained embryos.

### Study outcomes and variables

The primary outcome of the present study was the chemical pregnancy rate, and the secondary outcomes were the clinical pregnancy rate and the status of vaginal lactobacillary flora. Study variables were collected from gynecology and embryology records. We categorized study variables into 2 groups: a) baseline variables, including demographic characteristics (age and body mass index), infertility history (infertility duration, and baseline level of anti-Mullerian hormone), and ART cycle characteristics (the number of retrieved oocytes, transferred embryos, and the fertilization rate) and b) outcome variables.

The fertilization rate is defined as the total number of zygotes with 2 pronuclei divided by the total number of injected Metaphase II oocytes. The chemical pregnancy rate was evaluated 2 wk after ET and was defined as the ratio of the number of participants with a positive beta-human chorionic gonadotropin test to the total number of participants who underwent ET. Clinical pregnancy was diagnosed by visualizing fetal heartbeats during an ultrasound assessment (1).

#### Determination of the status of vaginal lactobacillary flora

The status of vaginal lactobacillary flora was examined before the ET, 2 wk after the intervention. A swab was used to collect vaginal fluid samples from the posterior vaginal wall during a standard vaginal speculum examination, performed by a gynecology resident who was unaware of the type of interventions. The samples were smeared on glass slides, air-dried, and gram-stained in the laboratory. Each slide had a unique code to hide the participant's identity and the type of interventions from laboratory staff. A laboratory technician, who was also blinded, examined the slides under the light microscope (100X) and classified the status of vaginal lactobacillary flora as positive or negative.

### Ethical considerations

All participants provided informed written consent before enrollment. Also, the study was designed and performed according to the Declaration of Helsinki. The study was approved by the Research Ethics Committees of Imam Khomeini hospital, Tehran University of Medical Sciences, Tehran, Iran (Code: IR.TUMS.IKHC.REC.1399.274). The study protocol was also registered while recruiting participants in the Iranian Registry of Clinical Trials (Registration date: November 10, 2020; Last update: April 03, 2024).

### Statistical analysis

All analyses were conducted using SPSS Statistics 23.0 (IBM Corp., Armonk, NY, USA) and graphs were designed by GraphPad Prism 9.5.1. (528) (GraphPad Software, San Diego, California USA). The normal distribution of data was tested by the Kolmogorov-Smirnov test. The categorical data were expressed as frequency and percentage, and quantitative data were expressed as mean ± SD if data has a normal distribution and median and rank in case of non-normal distribution. Chemical and clinical pregnancy rates were reported both as absolute (number and percentage) and relative effect sizes (risk ratio [RR]). Categorical data were analyzed using the Chi-squared test and quantitative data by *t* test (normal distribution) and Mann-Whitney test (non-normal distribution). 95% confidence interval (95% CI) was reported for each variable. P < 0.05 was considered as significant.

## 3. Results

**Figure 1:**
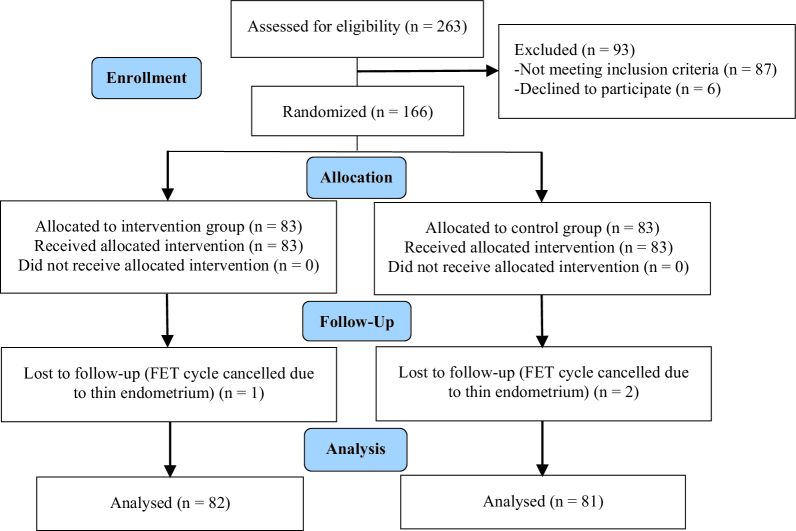
Study flow diagram. FET: Frozen embryo transfer.

Initially, 263 women were enrolled in the study. A total of 93 participants were excluded due to not meeting the inclusion criteria (87 cases) or declining to participate (6 cases). The remaining participants were randomly assigned to the intervention and control groups (n = 83/each). From the control group, 2 cases were excluded because of FET cycle cancelation due to unresponsive (thin) endometrium. In the intervention group, 1 participant was excluded due to the same reason (Figure 1).

Table I shows the baseline characteristics of the participants. No significant differences were observed between the groups in terms of demographic characteristics, infertility history, and ART cycle characteristics.

**Figure 2:**
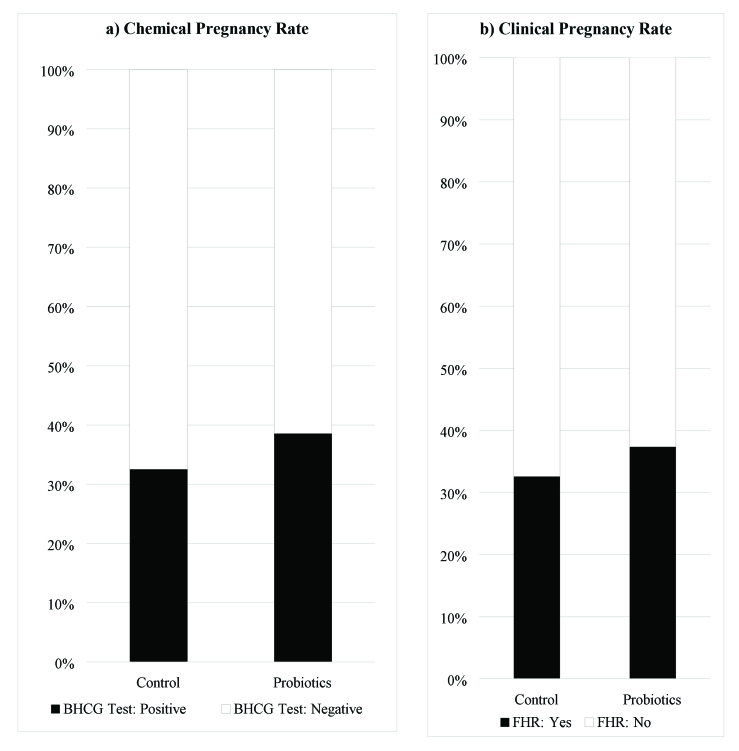
Comparison of rates of A) Chemical and B) Pregnancy rate between the groups. BHCG: Beta human chorionic gonadotropin, FHR: Fetal heart rate.

Figure 2 shows the chemical and clinical pregnancy rates in the 2 groups. The probiotics group had slightly higher rates than the control group, but the differences were not statistically significant. The chemical pregnancy rate was 39.02% in the probiotics group and 33.33% in the control group (RR: 1.71, 95% CI: 0.77–1.76; p: 0.449). The clinical pregnancy rate was 37.80% in the probiotics group and 33.33% in the control group (RR: 1.14, 95% CI: 0.76–1.74; p: 0.623).

**Figure 3:**
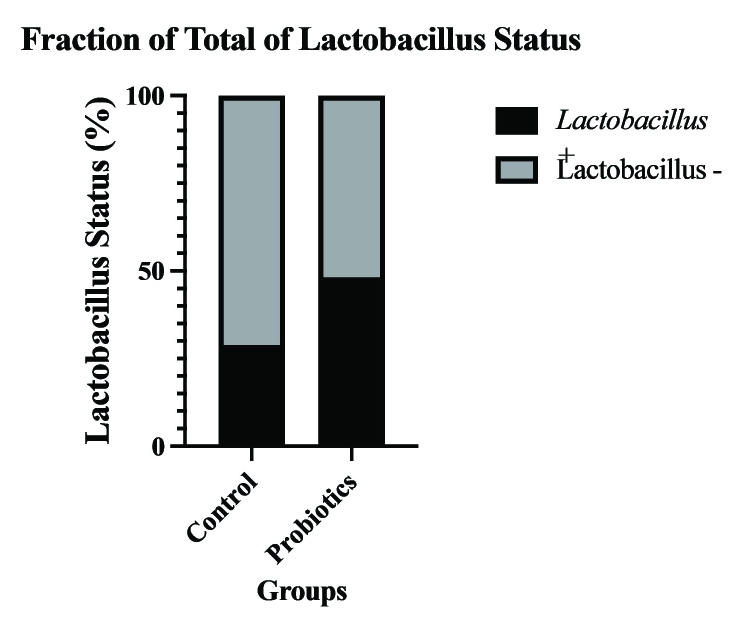
Comparison of pregnancy rate between the groups according to the status of vaginal lactobacillary flora.

Figure 3 shows the percentage of participants who had a positive *Lactobacillus* status in the 2 groups. The probiotics group had a significantly higher percentage than the control group (62.90% vs. 37.10%, p = 0.011). We also compared the pregnancy rates among the participants who received probiotics and had a positive or negative *Lactobacillus* status, and those who did not receive probiotics and had a positive *Lactobacillus* status. The pregnancy rate was highest in participants who received probiotics and had a* Lactobacillus*-positive status (42.5%). However, these differences were not statistically significant. Detailed results are shown in table II.

**Table 1:** Baseline characteristics


**Demographic characteristics**		**Probiotic (n = 82)**	**Control (n = 81)**	**95% CI**	**P-value**
**Age (yr) ${\;}^{\text{a}}$ **		32.26 ± 4.91	32.55 ± 4.86	-1.00 to 1.59	0.651
**BMI (kg/m^2^) ${\;}^{\text{a}}$ **		24.63 ± 2.46	25.03 ± 2.43	-0.34 to 1.14	0.287
**Infertility history**					
	**Infertility duration ${\;}^{\text{a}}$ **	7.85 ± 3.18	8.21 ± 2.97	-0.58 to 1.28	0.456
	**AMH ${\;}^{\text{a}}$ **	2.57 ± 0.61	2.45 ± 0.63	-0.30 to 0.07	0.218
**ART characteristics**					
	**No. oocytes retrieved ${\;}^{\text{a}}$ **	9.16 ± 3.18	8.65 ± 3.21	-1.47 to 0.46	0.304
	**No. of transferred embryos ${\;}^{\text{b}}$ **	2.39 ± 0.49 (2 [2–3])	2.48 ± 0.50 (2 [2–3])	-	0.348
	**Fertilization rate ${\;}^{\text{a}}$ **	52.09 ± 24.45	54.79 ± 26.18	-4.97 to 10.37	0.488
a) Data presented as the Mean ± standard deviation, Student *t* test. b) Data presented as Mean ± SD (MD, IQR), Mann-Whitney. BMI: Body mass index, AMH: Anti-Mullerian hormone, ART: Assisted reproductive technologies, CI: Confidence interval					

**Table 2:** The status of vaginal lactobacillary flora and its relationship with chemical pregnancy rate


**Groups**	***Lactobacillus*** ** flora status**	**No. of cases**	**Chemical pregnancy rate**	**RR**	**95% CI**	**P-value**
	Negative	58 (71.60)	19 (34.78)			
**Control (n = 81)**	Positive	23 (28.39)	8 (32.76)	1.06	0.52–1.97	0.861
		Negative	43 (52.43)				15 (34.88)			
**Probiotics (n = 82)**	Positive	39 (47.56)	17 (42.5)	1.25	0.72–2.15	0.419
		Negative	101 (61.96)				34 (33.66)			
**Whole sample (n = 163)**	Positive	62 (38.03)	25 (40.32)	1.19	0.79–1.78	0.390
	Data presented as number (%) and analysis were based on Chi-square test. RR: Risk ratio, CI: Confidence interval						

## 4. Discussion

This study aimed to evaluate the effects of intravaginal probiotic administration on the rates of chemical and clinical pregnancy in RIF cases undergoing FET. The main finding of this study was that the probiotic group had a slightly higher pregnancy rate than the control group, but the difference was not statistically significant. This suggests that empirical treatment with intravaginal probiotics in asymptomatic RIF cases before ET still has a long way to go.

Another finding of this study was that the probiotic group had a significantly higher percentage of participants who had a positive *Lactobacillus* status than the control group. This indicates that intravaginal probiotics can effectively modulate the vaginal microbiota and increase the prevalence of *Lactobacillus*, which is considered beneficial for reproductive health. However, the pregnancy rate was not significantly different among the participants who received probiotics and had a positive or negative *Lactobacillus* status, and those who did not receive probiotics and had a positive *Lactobacillus* status. The pregnancy rate was highest in participants who received probiotics and had a *Lactobacillus*-positive status (42.5%), but this difference was not statistically significant. This implies that other factors besides *Lactobacillus* status may influence the pregnancy outcome in RIF cases. Also, it might be caused by the small sample size of the study.

The present study is the first clinical trial evaluating the effect of vaginal probiotic supplementation in RIF cases. The different designs, populations, interventions, and outcomes of some of the previous similar studies limit the comparability of their results to our findings. For example, some studies used oral and others used vaginal probiotics, some studies included cases with different indications for IVF, such as polycystic ovary syndrome or male factor infertility, instead of RIF, and some studies measured non-reproductive outcomes like vaginal health parameters, *lactobacillus* colonization, or vaginal microbiota composition instead of the ART or pregnancy outcomes (19–21).

Among the previous studies, 4 studies assessed the effect of probiotic therapy in infertile females on ET outcomes (15–17, 22). Our finding is consistent with the first study on this subject, which found no effect of intravaginal probiotics on the pregnancy rate (16). These results indicate that the vaginal microbiome may not be a major determinant of implantation success in IVF cycles and that probiotics may not be able to modify the vaginal microbiome sufficiently to enhance endometrial receptivity and embryo survival.

However, our finding is in contrast with the studies that reported positive effects of probiotics on pregnancy rates in IVF cycles (15, 17, 22). It has been shown in a study that oral administration of *L. salivarius* CECT5713 increased the pregnancy rate in women with reproductive failure, and probiotic administration ameliorated crucial biochemical, microbiological, and immunological parameters in women who become pregnant (22). Another study found that intravaginal administration of lactobacilli reduced the miscarriage rate and increased the live birth rate in FET cycles, especially in women with bacterial vaginosis (BV) and in the blastocyst transfer group (15). A similar study in Iranian population also found that intravaginal administration of LactoVag improved the pregnancy rate in FET cycles, specifically in women who received grade A fetuses during ET (17). These results suggest that probiotics may have a role in improving the vaginal health and the quality of life of IVF cases and that probiotics may interact with other factors, such as the oral route, the reproductive failure, the bacterial vaginosis, the blastocyst stage, and the embryo quality, to influence the pregnancy rates in IVF cycles.

Our study also adds to the growing evidence that probiotic lactobacilli can effectively modulate the vaginal microbiota and increase the prevalence of *Lactobacillus*, which is considered beneficial for reproductive health. A systematic review and meta-analysis have demonstrated that intravaginal probiotics can restore and maintain normal vaginal flora, characterized by a high number and diversity of lactobacilli, a low vaginal pH, and a reduced presence of pathogenic bacteria (9). However, the effects of probiotics supplementation on pregnancy rates in IVF cycles, depending on vaginal flora status are still inconclusive. Among the similar researches to our study, only 2 studies, investigated both the outcome of ET and vaginal microbiota after probiotic supplementation (15, 16). One of them exactly like our study examined the positivity of the vaginal flora in terms of lactobacilli (16). Contrary to our finding they showed that the intravaginal administration of probiotics did not affect the prevalence of positive vaginal lactobacillary status. Furthermore, the positive vaginal lactobacillary status during oocyte retrieval or ET did not improve the pregnancy rates. The other study did not examine the presence of lactobacilli alone and examined the vaginal flora for BV (15). They found that among the women who were diagnosed with BV, the probiotic group had higher clinical pregnancy and live birth rates, although not statistically significant. They suggested that the probiotic supplementation may have reduced the adverse effects of BV on implantation and pregnancy rates. However, they also acknowledged that the small sample size in the subgroup analysis may have limited their power to detect small differences.

One of the strengths of this study was that it was the first randomized clinical trial to specifically investigate the effects of intravaginal probiotics on pregnancy rates in RIF cases undergoing FET. Another strength was that it used a standardized and validated probiotic formulation that contained high amounts of *Lactobacillus* strains, which are known to be beneficial for vaginal health and embryo implantation. Moreover, this study used a rigorous methodology, including randomization, blinding, and intention-to-treat analysis, to minimize the risk of bias and confounding.

However, this study also had some limitations that should be acknowledged. First, as mentioned above, the sample size may have been insufficient to detect a small effect of probiotics on pregnancy rates. Second, we could not follow up with the participants until the delivery, so it was not possible to evaluate the effects of probiotics on the perinatal outcomes, such as the preterm birth, the low birth weight, or the neonatal infections.

## 5. Conclusion

In conclusion, this study showed that intravaginal probiotic administration did not significantly improve the pregnancy rates in RIF cases undergoing FET. Further studies are needed to explore the optimal dose, duration, and timing of probiotic administration, as well as the mechanisms of action and the potential adverse effects of probiotics on the vaginal microbiome and the implantation process. Probiotics may still have a role as an adjunctive therapy for improving the vaginal health and the quality of life of IVF cases, but their routine use for enhancing pregnancy rates is not supported by the current study.

##  Data availability 

Data supporting the findings of this study are available upon reasonable request from the corresponding author.

##  Author contributions

All authors contributed to the study conceptualization and commented on previous versions of the manuscript. All authors read and approved the final manuscript. Detailed contribution statement: MNJ: Study design, supervision, data collection, study methodology, and interpretation of results; FH: Data collection, interpretation of results, and manuscript editing; MM: Data collection and manuscript review; MA: Data analyses and manuscript original draft preparation; SZ: Data collection and manuscript editing.

##  Conflict of Interest

The authors declare that there is no conflict of interest.
